# Performance and host association of spotted lanternfly (*Lycorma delicatula)* among common woody ornamentals

**DOI:** 10.1038/s41598-021-95376-x

**Published:** 2021-08-04

**Authors:** Osariyekemwen Uyi, Joseph A. Keller, Emelie Swackhamer, Kelli Hoover

**Affiliations:** 1 Department of Entomology, Pennsylvania State University, 501 ASI Building, University Park, PA 16802 USA; 2 Department of Animal and Environmental Biology, University of Benin, P.M.B. 1154, Benin City, Nigeria; 3 Horticulture Educator, Penn State Extension Montgomery, Collegeville, PA 19426 USA

**Keywords:** Entomology, Invasive species

## Abstract

*Lycorma delicatula* (spotted lanternfly) has a broad host range with a strong preference for the invasive host plant from its native range, tree of heaven (*Ailanthus altissima);* it had long been speculated that *L. delicatula* could not develop or reproduce without access to tree of heaven*.* In 2019, we found that this assumption was incorrect, but fitness was reduced in the absence of *A. altissima* in that the number of egg masses laid was dramatically fewer for insects reared on suitable non-*A. altissima* host plants that had recently been established. We hypothesized that longer established, larger trees (of the same species) would improve the fitness of *L. delicatula* in the absence of tree of heaven. In spring 2020, we examined insect performance with and without access to *A. altissima* by tracking development, survival, host tree association and oviposition in large enclosures with trees planted two years prior to the study. Each enclosure included one each of *Juglans nigra*, *Salix babylonica* and *Acer saccharinum* along with either one *A. altissima* or one *Betula nigra;* these trees had twice the diameter of the same trees the previous year. We reared nymphs with and without access to *A. altissima*, released them into the corresponding large enclosures as third instars, and monitored them from early July 2020 through November 2020. We also determined whether lack of access to *A. altissima* by parents of *L. delicatula* have any fitness effects on offspring performance. To ensure adequate adult populations for comparing fecundity between treatments, third instars were released into the multi-tree enclosures due to high mortality in earlier instars that occurred in a similar study in 2019. Insect survival was higher and development faster with access to *A. altissima*. Third and fourth instar nymphs were most frequently observed on *A. altissima* when it was present, while adults were equally associated with *A. saccharinum* and *A. altissima*. In the absence of *A. altissima*, nymphs were most frequently found on *S. babylonica,* while adults were most often on *A. saccharinum*. Females with access to *A. altissima* deposited nearly 7-fold more egg masses than those without access to *A. altissima,* which is consistent with the difference in egg mass numbers between the two treatments the previous year; thus, our hypothesis was rejected. The offspring of parents that had been reared without access to *A. altissima* showed similar survival and development time from egg to adult as offspring from parents that never had access to *A. altissima*. These findings suggest that managers need to be aware that even in the absence of *A. altissima* in the landscape, several hardwood host trees can be utilized by *L. delicatula* to develop and reproduce, but fitness without *A. altissima* is likely to still be reduced.

## Introduction

Since 2014, the spotted lanternfly (SLF), *Lycorma delicatula* (White) (Hemiptera: Fulgoridae), an exotic planthopper, has invaded nine states in the Northeast, mid-West, and mid-Atlantic regions of the U.S. Native to China, Taiwan and Vietnam, *L. delicatula* has also expanded its range to include South Korea and Japan^1–4^. Following its initial detection in Berks County, Pennsylvania^1^, *L. delicatula* has spread to New Jersey, New York, Virginia, Maryland, Delaware, West Virginia, Connecticut, and Ohio^4,5^. The invasion success of this pest may be partly due to its broad host range^2^, apparent capacity for dispersal^6,7^ and its potential to occupy a wide range of climatic conditions and ecosystems, especially disturbed habitats where the preferred host *Ailanthus altissima* (Mill.) Swingle (Simaroubaceae) (tree-of-heaven) is abundant^3,8,9^.

As a generalist phloem sap feeder, *L. delicatula* has proven to be a prominent pest in forest and agricultural ecosystems and a nuisance pest in suburban landscapes, causing significant economic losses to vineyards, nurseries, and sawmills in the invaded regions^4,10^. For the ornamentals and forest products industries, the major economic impacts are primarily due to the cost of best management practices to comply with quarantine restrictions, and in some cases, reduced sales^10,11^. *Lycorma delicatula* is projected to cause $42.6 million in damages annually if it spreads across Pennsylvania^11^. Although studies on the impacts of feeding by *L. delicatula* on host plant health are in their infancy, phloem feeding by nymphs and adults is known to cause physiological stress in young stems that may in turn cause infested branches to wilt, lose vigor or die following heavy infestations^10,12^. Feeding by *L. delicatula* results in the production of copious amounts of honeydew, which promotes the growth of sooty mold on plants below feeding sites, impeding photosynthesis of affected plants^13–16^. Damage caused by *L. delicatula* decreases grapevine growth, reducing crop yield by up to 90% and sometimes killing the vines (*Vitis* spp. L. [Vitales: Vitaceae])^10,12^. Additionally, *L. delicatula* poses a threat to the growth of several hardwood trees, including silver maple (*Acer saccharinum* L. [Sapindaceae]), red maple (*A. rubrum* L. [Sapindaceae]), black walnut (*Juglans nigra* L. [Juglandaceae]), weeping willow (*Salix babylonica* L. [Salicaceae]), river birch (*Betula nigra* L. [Betulaceae]) and tulip tree (*Liriodendron tulipifera* L. [Magnoliaceae])^1,2,17–19^.

**Figure 1:**
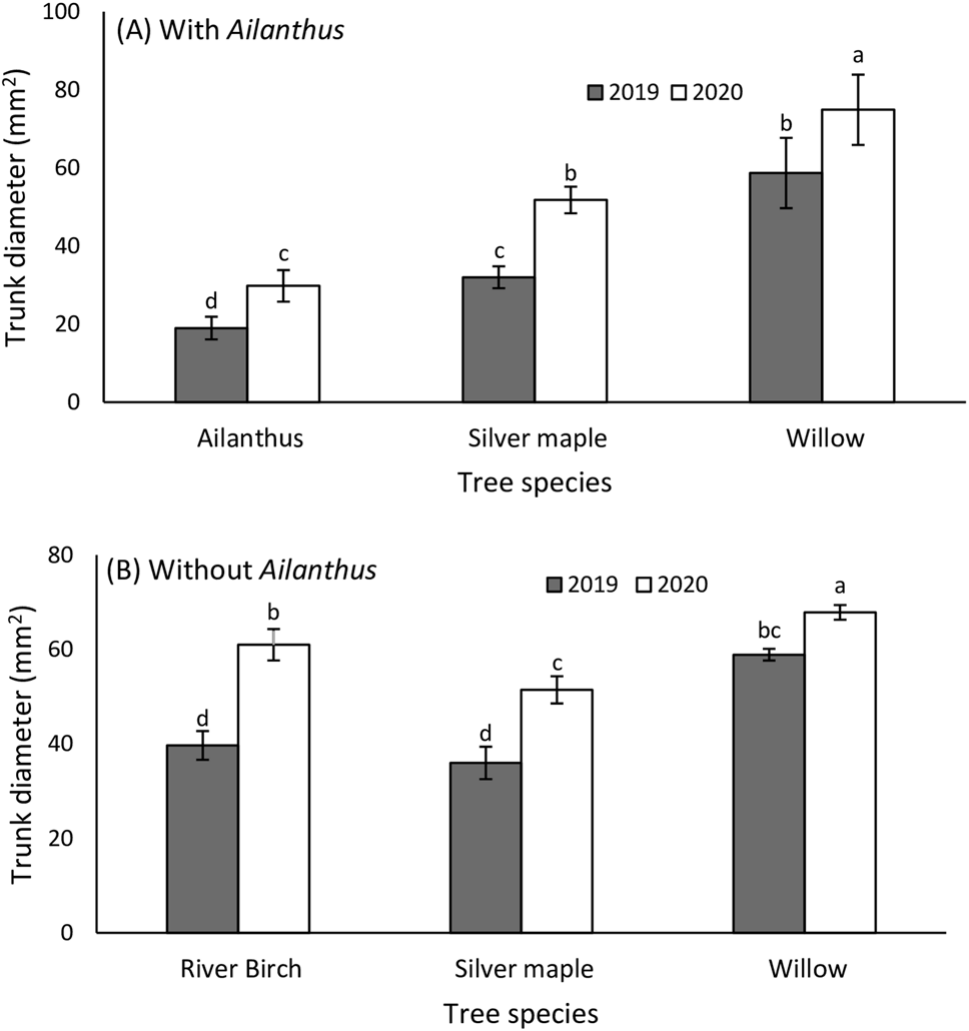
Mean (± SE) trunk diameter (mm) of tree species in enclosures with *Ailanthus* (**A**) and without *Ailanthus* (**B**) (*n* = 5 enclosures) in 2019 and 2020. Measurements were made on October 31, 2019 and November 3, 2020. Means within a column followed by different letters are significantly different (Bonferroni post hoc test: *P* < 0.05). Black walnut trees were not included in the analysis because most died during the 2019 studies and had to be replaced in spring of 2020.

*Lycorma delicatula* prefers *A*. *altissima* in both its native and invasive ranges, though it can utilize over 103 plant species across 33 families^4,17,18^. In North America, feeding has been observed on 56 plant species, which include native, cultivated, and nonnative species^18^. The rapid spread of this pest is likely facilitated by the prevalence of *A. altissima*, in addition to other suitable host plant species^18^. Despite the broad host range of *L. delicatula*, not much is known about its host preferences and relative performance when feeding on common woody ornamentals but Murman et al.^20^ reported that eight species were able to support the development of *L. delicatula* from first instars to adulthood. Our previous study in 2019^19^ showed that this pest can complete development and reproduce on young weeping willow (*S. babylonica*), silver maple (*A. saccharinum* L) and river birch (*B. nigra*) without access to *A. altissima*. But, development from egg to adult was delayed by one week and the number of egg masses laid by females was significantly fewer (6.7-fold) in enclosures with no access to *A. altissima*. We hypothesized however, that *L. delicatula* without access to *A. altissima* can develop and produce as many eggs as those with access to *A. altissima* if provided trees that have been established longer and are bigger than the trees they fed on in the previous year. As a voracious sap feeder, *L. delicatula* appears to prefer larger trees as an adult (personal observations). Thus, in 2020, we investigated the fitness and host association of *L. delicatula* among planted common woody ornamentals using trees that were 2-fold larger in diameter in 2020 than in 2019 (see tree size measurement details in “Materials and methods” section, Fig. 1.). A further objective of this study was to determine whether offspring (F_1_ generation) from parents that were reared from egg to adult without access to *A. altissima* suffer any lingering fitness effects as they develop into adults.

## Materials and methods

### Origin and maintenance of trees and insect cultures

In Macungie, Berks County, PA, we set up ten 5.76 m^2^ plots in mid-September 2018; five plots each were randomly allocated to two treatments: ‘with *Ailanthus*’ and ‘without *Ailanthus*’^19^. All plots contained one *S. babylonica*, one *A*. *saccharinum*, and one *J. nigra* spaced 1 m apart; in addition, the ‘with *Ailanthus*’ plots were also planted with one *A. altissima*, while the ‘without *Ailanthus*’ plots contained one river birch (*B. nigra*) in place of *A. altissima*. We purchased the *Salix babylonica* and *B. nigra* as 15-gallon potted trees (~ 200 cm tall) at New Hanover Gardens (Perkiomenville, PA), and *A*. *saccharinum* as 7-gallon (~ 150 cm tall) and *J. nigra* as 2-gallon (~ 90 cm tall) trees from Octoraro Native Plant Nursery (Kirk, PA). Since *A. altissima* is a ubiquitous invasive plant*,* small trees (~ 90 cm tall) were dug up and transplanted from a local property with the owner’s permission. We selected these species because they are common in this region of the U.S. and are often used as hosts trees by *L. delicatula* in Pennsylvania. *Lycorma delicatula* were confined to each plot using a rectangular screen enclosure made of PAK25 Anti-Insect Mesh (Hummert International, MO) attached to a frame (3 × 2.4 × 2.4 m) made of galvanized steel tubes (38 mm diameter) at each corner to prevent collapse during windy thunderstorms. To obtain access to each enclosure we installed a 1.8 m zipper on one side. To prevent *L. delicatula* nymphs or adults from escaping from the enclosures, sandbags (7.6 × 99 cm) were sewn into the bottom edge of the enclosure to weigh down the sides. We installed drip irrigation connected to the well on the property. In the early spring, trees were pruned to minimize crowding.

In January 2020, egg masses of *L. delicatula* were field collected in Allentown, PA and kept in two pop-up cages (90 × 60 × 60 cm) under ambient environmental conditions in an unheated greenhouse in Macungie, PA. In early May, egg masses were transferred to 20 pop-up cages (90 × 60 × 60 cm) containing potted host plants and housed under a canopy at the experimental site. Here, the pop-up cages were used for rearing newly hatched nymphs until they reached the third instar. In this way, all plants received direct sunlight for part, but not all, of the day. These pop-up cages were divided between two treatments: ‘with *Ailanthus*’ (10 cages) and ‘without *Ailanthus*’ (10 cages). Pop-up cages ‘with *Ailanthus’* contained one potted ~ 60 cm tall *A. altissima* (planted from field-collected seeds), one potted ~ 45 cm tall *V. vinifera* (cv. Cabernet-Franc; Hermann J. Wiemer Vineyard, Dundee, NY), one potted ~ 45 cm tall strawberry and one potted ~ 45 cm tall sunflower, while pop-up cages ‘without *Ailanthus*’ contained one potted ~ 45 cm tall *V. vinifera*, one strawberry and one sunflower. Neonates in both treatments were reared to third instar until release into each multi-tree enclosure.

### Experiment I: *Lycorma delicatula* survival, development and host plant associations

Between July 7 and July 10, 2020, 120 one- to three-day old third instars were released into each multi-tree enclosure. Enclosures ‘with *Ailanthus*’ received nymphs with prior access to *A. altissima* from the pop-up cages with potted *A. altissima* (see details above) and vice versa^19^. To maximize the availability of adults for comparing fecundity between the two treatments, we released third instars into the enclosures to avoid the early instar mortality evident in our 2019 study^19^ in which we found that *L. delicatula* can be reared from first instar to adult without access to *A. altissima*. Each week we monitored plots for survival, life stage, and which host tree the insects were found on by counting the numbers of nymphs (from July 15, 2020 to September 2, 2020) and adults (August 12, 2020 to November 4, 2020) present or seen on each host tree species and on other surfaces in each enclosure. After reaching adulthood we placed wooden planks and red maple logs in the enclosures to provide additional oviposition material and began monitoring and recording for oviposition weekly through November 4, 2020 when a hard freeze killed all remaining adults. To document growth between 2019 and 2020, trunk diameter measurements were made on October 31, 2019 and November 3, 2020, and we compared trunk diameter of trees by species between 2019 and 2020. Trunk diameter measurements for *J. nigra* were not available because most of these trees had died during the 2019 studies and were replaced in spring of 2020. For both treatments, trunk diameter differed significantly by year (‘with *Ailanthus*’: *F*_1,29_ = 79.27; *P* = 0.0001; ‘without *Ailanthus*’: *F*_1,29_ = 40.84; *P* = 0.0001) and tree species (‘with *Ailanthus*’: *F*_2,29_ = 45.47; *P* = 0.0001; ‘without *Ailanthus*’: *F*_2,29_ = 101.87; *P* = 0.0001). There were no significant tree species × year interactions (‘with *Ailanthus*’: *F*_2,29_ = 1.15; *P* = 0.334; ‘without *Ailanthus*’: *F*_2,29_ = 2.25; *P* = 0.070). Trees used in 2020 were on average 26.0 ± 2.3% larger than the same trees were in 2019 (Fig. 1A,B).

### Experiment II: Performance of *L. delicatula* offspring

To compare fitness of offspring from parents that did or did not have access to *A. altissima* trees during development from egg to adult, we collected egg masses from the prior experiment (as above) conducted in 2019^19^. In early-June of 2020 these egg masses were carefully collected from the trees in each multi-tree plot, placed in Petri dishes and held in pop-up cages (90 × 60 × 60 cm) containing two potted ~ 60 cm tall *A. altissima* trees (planted from seeds), with 4 replicates per treatment. Here, the pop-up cages were used to rear the newly hatched nymphs until adult emergence. All pop-up cages were placed in open field conditions in the same location but were sheltered from direct rainfall. Trees were watered thrice per week and replaced once per month with fresh potted *A. altissima* trees. Weekly, we recorded survival and development of *L. delicatula* by counting the numbers of nymphs and adults present and seen in each cage.

### Statistical analysis

To evaluate the effect of the presence or absence of *A. altissima* on the survival of *L. delicatula* in Experiments I and II, we fit a generalized linear mixed model with a binomial error distribution and logit link function using the *glmmTMB* function^19^ in R^21,22^. Our model included treatment, date of observation, and the interaction between these two factors as predictors, and cage as a random effect to account for repeated observations. Similarly, to assess the effect of *A. altissima* presence on the timing at which individuals reached the fourth instar and adulthood in Experiments I and II, we fit a GLM mixed effects model with a binomial error distribution and a logit link function. We predicted the proportion of individuals in each cage that had reached the fourth instar and adult stages based on the date, treatment (with or without *A. altissima*) and interaction of these two factors. We again included cage as a random effect in the model to account for repeated observations. We assessed the significance of the treatment by conducting a likelihood ratio test to compare the full model against a reduced model, excluding treatment as a predictor. Repeated measures GLM ANOVA was used to compare the host plant association (i.e., the proportion of nymphs and adults of *L. delicatula* on a given tree species) of *L. delicatula* in the presence or absence of *A. altissima* using SPSS version 20.0 (IBM, SPSS Inc. Chicago, IL). If the overall model was significant, differences between trees was determined using the Bonferroni post hoc test. The Mann Whitney U test was used to compare the number of eggs masses between treatments using SPSS version 20.0. *Juglans nigra* trees were not included in the analysis because most died during the 2019 studies and had to be replaced in spring of 2020.

### IUCN policy statement

Collection of plant material was done in compliance with relevant institutional, national, and international guidelines and legislation.

## Results

### Survival, development time, host association and egg mass count

**Figure 2:**
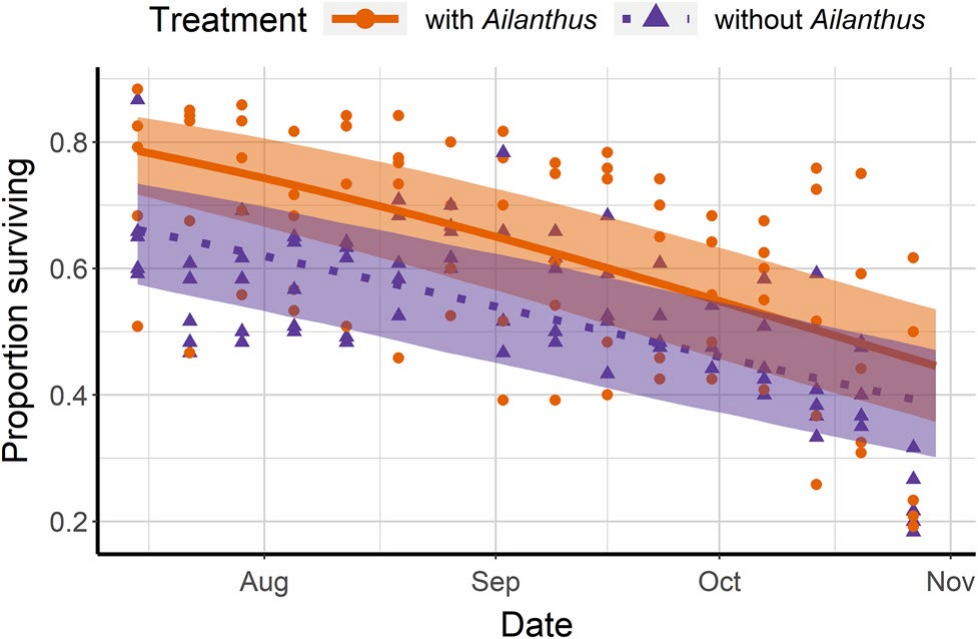
Proportion of *Lycorma delicatula* (nymphs and adults combined) that survived in enclosures with planted *Ailanthus altissima*, *Acer saccharinum*, *Juglans nigra* and *Salix babylonica* (‘*Ailanthus*,’ *n* = 5 enclosures) or enclosures with the same tree species except for the presence of *Betula nigra* in place of *A. altissima* (‘without *Ailanthus*,’ *n* = 5 enclosures) from July 15 to November 4, 2020. Fitted binomial regression lines are shown, with shaded areas indicating 95% confidence intervals for predicted regression means. In total, 120 third-instar nymphs were released in each enclosure and their numbers and host tree associations were recorded weekly.

**Figure 3:**
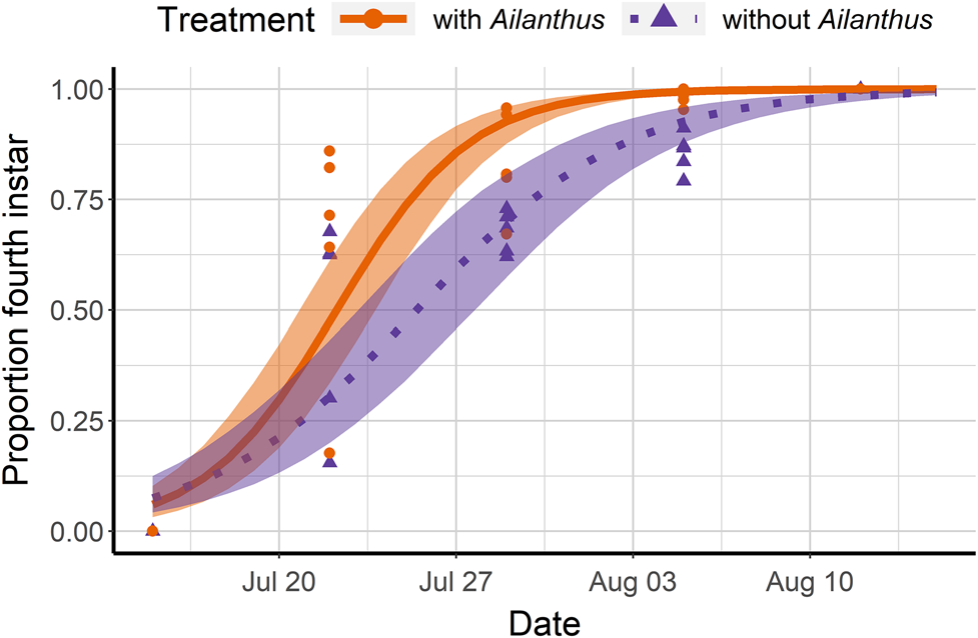
Proportion of *L. delicatula* that developed from the third to the fourth instar over time, with fitted logistic regression lines, in enclosures with and without *Ailanthus altissima*.

**Figure 4:**
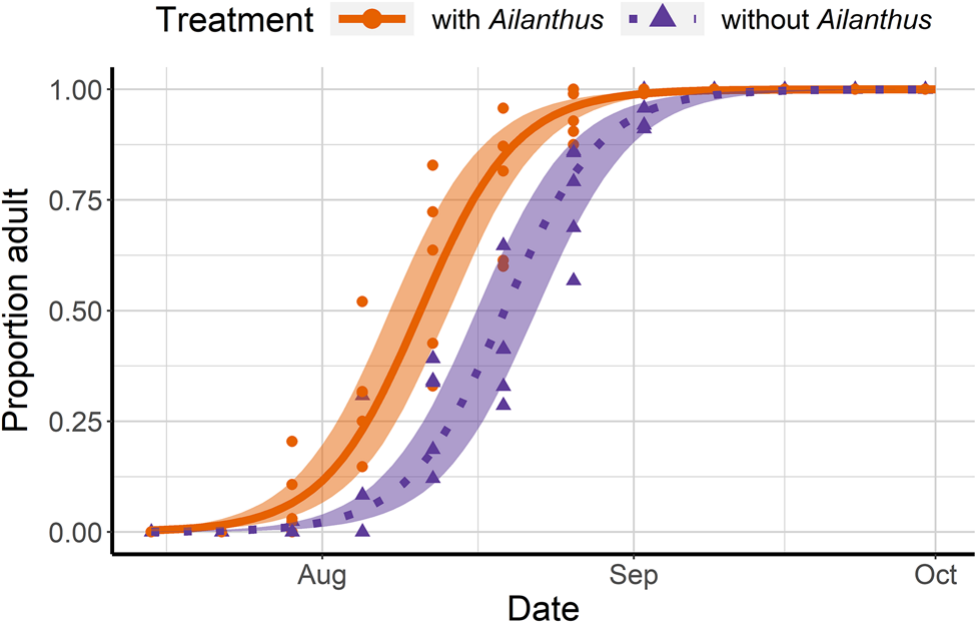
Proportion of *L. delicatula* that emerged as adults over time, with fitted logistic regression lines, for enclosures with and without *Ailanthus altissima*.

Following release into multi-tree enclosures, the proportion of individuals of *L. delicatula* (from third instar nymph to adult) that survived was approximately 10% higher in enclosures with *A. altissima* compared to the non-*Ailanthus* enclosures throughout the season (*χ*^*2*^ = 16.29, df = 1, *P* = 0.001; Fig. 2). Third instar nymphs in *A. altissima* enclosures developed slightly faster to fourth instar (*χ*^*2*^ = 56.26, df = 1, *P* = 0.001; Fig. 3); fitted logistic regression curves showed that 50% of third instars with access to *Ailanthus* reached the fourth instar 2.1 days earlier than those without *Ailanthus*. In cages containing *A. altissima* 50% of fourth instars reached adulthood 8.4 days earlier than those in enclosures without *A. altissima* (*χ*^*2*^ = 9.67; *P* = 0.001; Fig. 4).

**Figure 5:**
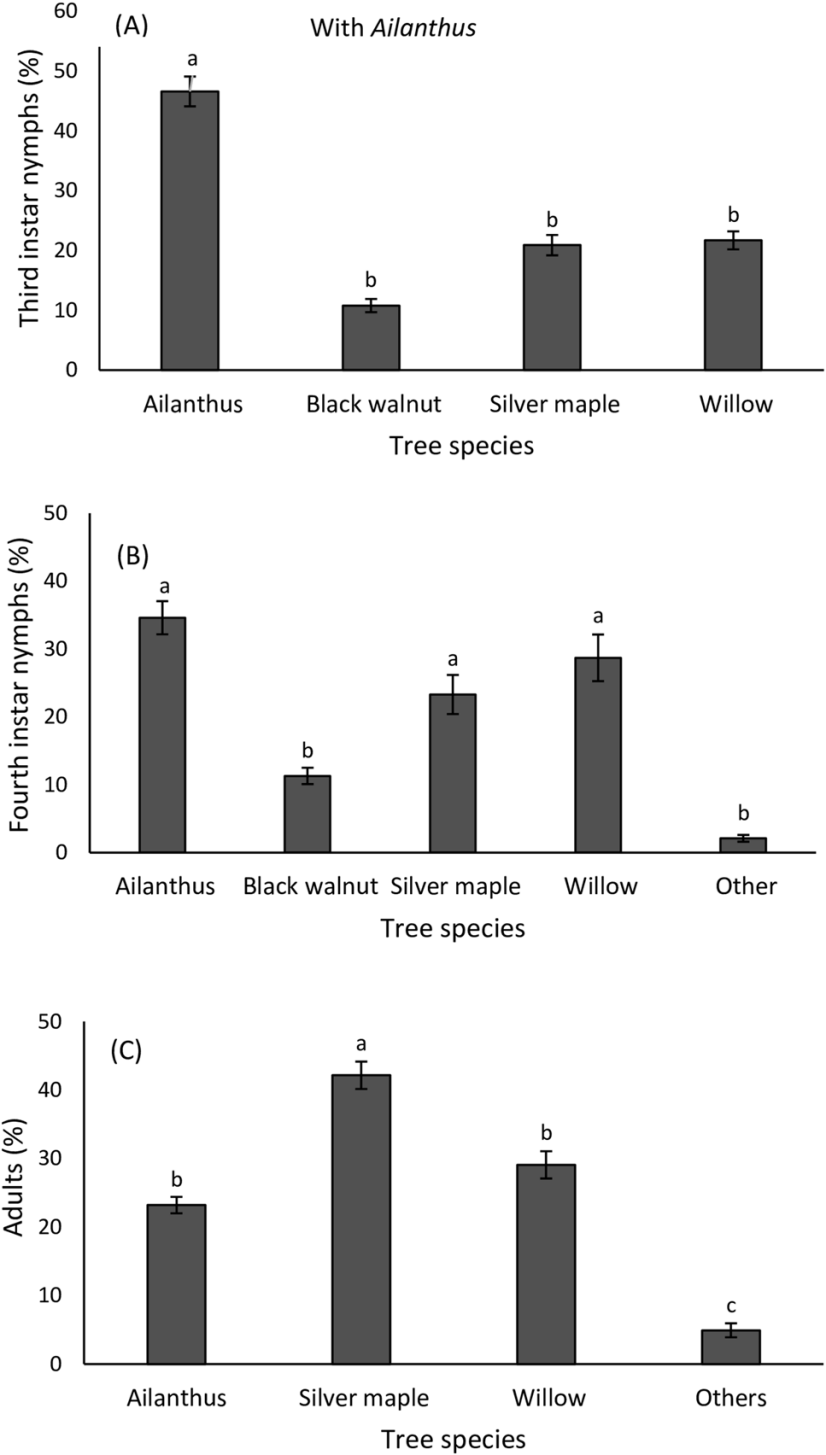
Mean (± SE) proportion of *Lycorma delicatula* third instars (**A**) fourth instars (**B**) and adults (**C**) present on each tree species in enclosures with *Ailanthus* (*Ailanthus altissima*), silver maple (*Acer saccharinum)*, black walnut (*Juglans nigra*) and willow (*Salix babylonica*) (*n* = 5 enclosures) from July 15 to November 4, 2020. Nymphs and adults found on annual plants (2%) within the enclosure or on enclosure walls (98%) are included as ‘Others.’ Means within a column followed by different letters are significantly different (Bonferroni post hoc test: *P* < 0.05). No adults were found on black walnut and no third instar nymphs were found on other surfaces.

**Figure 6:**
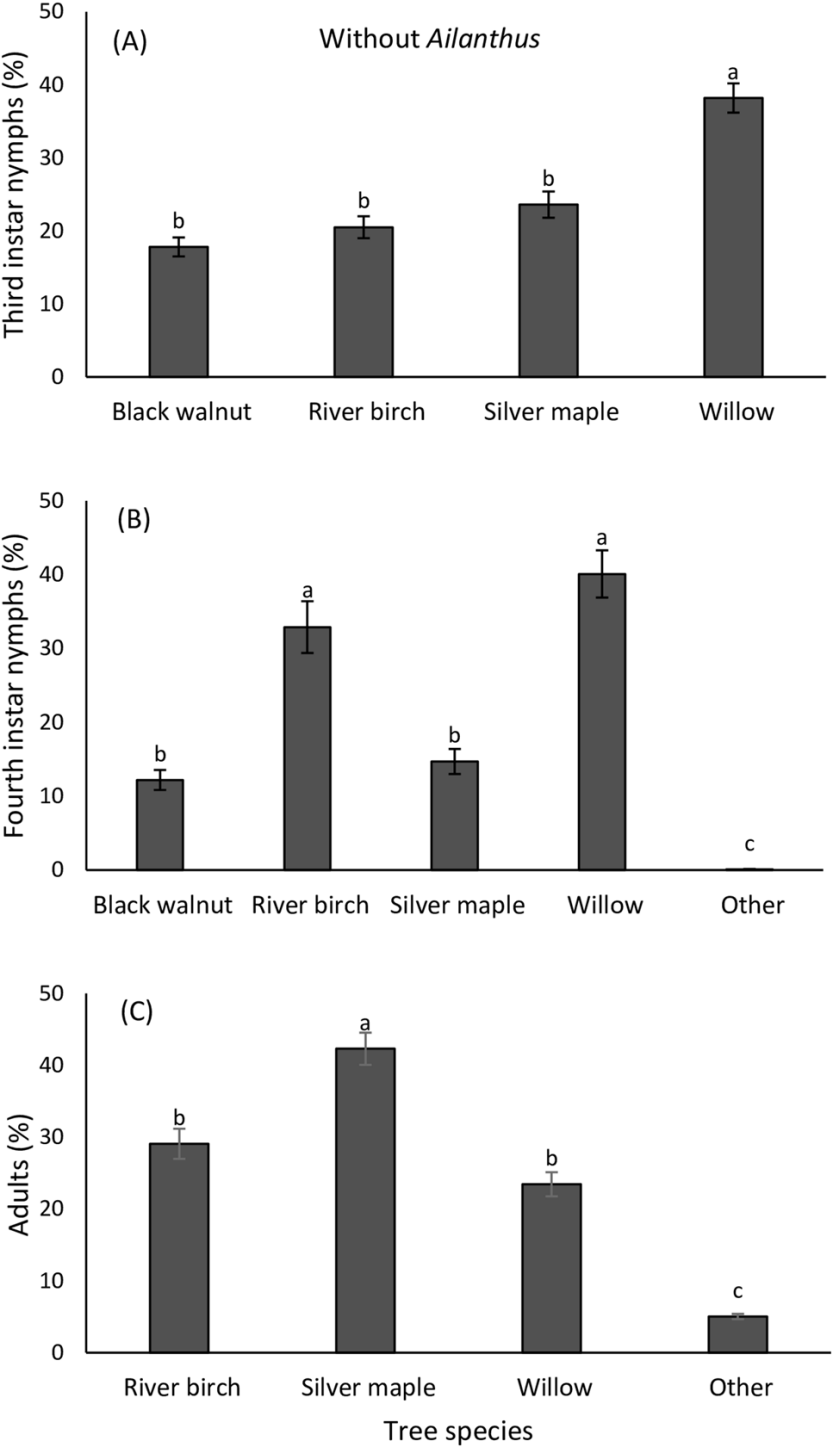
Mean (± SE) proportion of *Lycorma delicatula* third instars (**A**), fourth instars (**B**) and adults (**C**) present on each tree species in enclosures with silver maple (*Acer saccharinum*), black walnut (*Juglans nigra*)*,* willow (*Salix babylonica*) and river birch (*Betula nigra*) (without *Ailanthus altissima*, *n* = 5 enclosures) from July 15 to November 4, 2020. Nymphs and adults found on other plants (2%) within the enclosure or on enclosure walls (98%) are included as ‘Others.’ Means within a column followed by different letters are significantly different (Bonferroni post hoc test: *P* < 0.05). No adults were found on black walnut and no third instar nymphs were found on other surfaces.

Host plant association of third and fourth instar nymphs as well as adults in *A. altissima* enclosures were influenced by the available tree species but did not differ over time (Table 1; Fig. 5A–C). In enclosures with *A. altissima*, third instars were most frequently observed on *A. altissima* (Fig. 5A)*,* while fourth instars were found with equal frequency on *A. altissima*, *S. babylonica* and *A. saccharinum* (Fig. 5B). Adults were mostly observed on *A. saccharinum* despite the presence of *A. altissima* (Fig. 5C). In enclosures without *A. altissima*, host plant association of nymphs and adults differed as a function of tree species but did not differ over time (Table 2; Fig. 6A–C). In enclosures without *A. altissima*, third instars were most frequently observed on *S. babylonica* (Fig. 6A), while fourth instars were found with equal frequency on *S. babylonica* and *B. nigra* (Fig. 6B). Again, adults were most commonly associated with *A. saccharinum* (Fig. 6C).

**Table 1:** Repeated measures binomial GLM ANOVA for data on the effect of tree species and time (week) on the proportion of *Lycorma delicatula* nymphs and adults present on individual trees in cages where they had access to *Ailanthus altissima.*

Variable	Source of variation	df	MS	*F*-value	*P*-value
Third instars	Tree species	3	3495	11.20	**0.001**
	Time (week)	16	0.023	0.001	0.999
	Tree species × Time	9	388.3	1.24	0.301
	Error	59			
Fourth instars	Tree species	4	5218	20.68	**0.001**
	Time (week)	5	0.067	0.011	0.967
	Tree species × Time	20	414.2	1.84	0.061
	Error	100			
Adults	Tree species	3	14,347	13.45	**0.001**
	Time (week)	11	1.03	0.009	0.991
	Tree species × Time	33	358.9	2.98	0.063
	Error	176			

df, degrees of freedom; MS, mean squares.

Statistically significant values are indicated in bold.

**Table 2:** Repeated measures binomial GLM ANOVA for analysis of data on the effect of tree species and time (week) on the proportion of *Lycorma delicatula* nymphs and adults present on individual trees in cages without *Ailanthus altissima.*

Variable	Source of variation	df	MS	*F*-value	*P*-value
Third instars	Tree species	3	1241	10.42	**0.001**
	Time (week)	3	0.09	0.05	0.970
	Tree species × Time	9	292.3	2.45	0.057
	Error	59			
Fourth instars	Tree species	4	4071	8.22	**0.001**
	Time (week)	5	0.021	0.001	0.999
	Tree species × Time	20	342.5	1.42	0.129
	Error	100			
Adults	Tree species	3	14,352	13.05	**0.001**
	Time (week)	11	0.32	0.003	0.993
	Tree species × Time	33	3231	2.11	0.071
	Error	176			

df, degrees of freedom; MS, mean squares.

Statistically significant values are indicated in bold.

The number of egg masses laid by females was 6.8-fold higher in enclosures with *A. altissima* (‘with *Ailanthus*’: 19.20 ± 2.13 (mean ± SE) than ‘without *Ailanthus*’ 2.80 ± 0.42; *U* = 20.0, *P* = 0.040). Egg masses were first observed on September 30, 2020 in the presence of *A. altissima* and oviposition continued until November 4 when field temperature dropped below 0 °C, killing the remaining adults in both treatments. Oviposition occurred in all five of these enclosures for a total of 96 egg masses. Of these egg masses, 23 were laid on *A. altissima*, 21 on *A. saccharinum*, 30 on *S. babylonica*, 1 on *J. nigra*, and 14 and 7 on wooden planks and red maple logs placed in the enclosures, respectively. In enclosures without *A. altissima,* 14 egg masses were recorded, with at least one egg mass in each of the 5 enclosures. Oviposition in these cages occurred between October 14 and November 4, 2020. Of these 14 egg masses, one was on *B. nigra*, 2 on *S. babylonica*, 6 on *A. saccharinum*, 2 on planks and 3 on red maple logs.

### Offspring performance

After being released in pop-up cages with access to healthy *A. altissima,* the proportion of hatched offspring from eggs laid the previous fall did not differ as a function of parental diet (*χ*^*2*^ = 0.70, df = 1, *P* = 0.401). Development time (hatch to adults) also did not differ between offspring from parents with or without access to *A. altissima* (*χ*^*2*^ = 0.13, df = 1, *P* = 0.722).

## Discussion

*Lycorma delicatula* can complete development and reproduce without access to *A. altissima,* confirming our findings from the previous year^19^, and providing additional insight into host association of *L. delicatula* among common woody ornamental trees. Fitness of the insect was greater in the presence of *A. altissima.* Survival was higher, development time was faster, and the number of egg masses was 6.8-fold higher than for insects that lacked access to *A. altissima*. This study also showed that offspring from parents that had been reared without access to *A. altissima* did not suffer fitness effects into the next generation. We hypothesize that reduced egg mass production in cages without *Ailanthus* was caused by slower development resulting in a shorter time span between adult emergence and freezing-induced mortality during which adults can mate and lay eggs. However, in southern climates where freezing temperatures appear several months later in the year or not at all, lack of access to *A. altissima* may have less impact on fitness since there could be time for slower developing adults to continue oviposition into the early winter.

In this study, survival to adult was high for both treatments (with and without *Ailanthus*), although survival was slightly higher (10% difference) in the presence of *A. altissima*. By early September 2020, approximately 63% of the individuals released (with and without access to *Ailanthus*) were still alive. This is in contrast to our 2019 study where survival on younger, smaller trees was less than 20% for both treatments (with and without access to *Ailanthus*) by September 2 when most lanternflies were adults^19^; however, the previous study started with the release of newly hatched first instars. Lower survival in 2019 could have been due to early mortality of first and second instar nymphs and/or having access to smaller trees in 2019 than in 2020 in that larger and/or more vigorous trees may provide a greater volume of sap and nutrients over time.

The faster development time of *L. delicatula* in enclosures with *A. altissima* suggests that adults could reach sexual maturity faster in areas where *A. altissima* is abundant. Development time remains one of the most crucial fitness indicators of host plant nutritional quality and can influence insect survival, behavior and physiology^23^. For example, several studies have found that slow development in phytophagous insects can cause high mortality of immature stages in the field by exposing them to unfavorable environmental conditions for a longer time period^24,25^. A key fitness cost caused by prolonged development in *L. delicatula* was narrowing of the reproductive window for adults, which was reflected in markedly fewer eggs being laid before freezing temperatures killed the remaining adults^19^.

Third and fourth instar nymphs were mostly associated with *A. altissima* when it was present, however, more adults were found on *A. saccharinum* than on *A. altissima* when both tree species were present. This may occur when the pest has exhausted the ability of *A. altissima* to provide sufficient sap flow, which also coincides with the onset of senescence of this tree species in mid-September. In the absence of *A. altissima*, *B. nigra*, *A. saccharinum, J. nigra* and *S. babylonica* together appeared to provide sufficient nutrition for growth and reproduction, but sap flow may be lower in these species, which could explain the delayed development time in the absence of *A. altissima.* Preferred host trees (*A. altissima* and *V. vinifera*) with a high sap flow are known to support faster development and increased performance in *L. delicatula*^26–28^. However, we cannot rule out that if given access to mature and vigorous suitable hosts such as *A. saccharinum* or *A. rubrum*, sexual maturity could have occurred at the same rate as those with access to *A. altissima*. These two maple species are heavily utilized by *L. delicatula* adults in the field in the fall (D.D. Calvin et al. unpublished data). Although the trees in our study were significantly larger than they were the previous year, they were still smaller than trees that are selected by wild populations of *L. delicatula* adults in the field where profuse feeding is associated with reproductive maturation (pers. observations).

Several factors may influence feeding preference in *L. delicatula* ranging from host-tree bark characteristics, sap sugar content and flow rate to the presence or absence of defensive chemicals^26–28^. *L. delicatula* appears to sequester plant defensive chemicals such as alkaloids and quassinoids as a defense against predation, the timing of which may coincide with the onset of aposematic coloration in the fourth instar^26^. Plant architecture may also play a role in host selection; this pest is often observed feeding on hosts with trunks and branches that do not have thick bark. The trees used in our study were chosen because they are frequently infested in the field and do not have the thick bark that occurs on some trees, such as oaks, which are infrequent hosts^29^. *L. delicatula* has been reported to survive longer on, and prefer, host trees with a high concentration of sap sugars, similar to those produced by *A. altissima* and *V. vinifera*^28^. In prior work by Lee and colleagues^28^, the authors reasoned that the improved performance on and preference for *A. altissima* and *V. vinifera* is related to high concentrations of sucrose and glucose in *A. altissima* sap, and high concentrations of sucrose and fructose in *V. vinifera* sap. Ingestion of sap by fulgorids is passive because they lack a cibarium pump (musculature in the pre-oral cavity that pumps fluids from a food source)^17,18,20^. Several authors have noted that *L. delicatula* seems to prefer hosts that have high turgor pressure within the phloem vessels, allowing for ingestion of sap at a greater flow rate.

Although egg masses were laid on all host trees as well as planks and red maple logs, *S. babylonica*, *A. saccharinum*, and *A. altissima* were generally preferred for oviposition. Oviposition substrate selection can be vital to the reproductive success of insect herbivores. The preference-performance hypothesis (a.k.a. mother knows best hypothesis) predicts correlation between oviposition preference by the female parent and host suitability for offspring development^30,31^. However, it does not translate well to species like *L. delicatula* with immature stages that are highly mobile or instances where the adults and immature life stages feed on different hosts^32^. Although tree species and branch structure may play a role in the selection of sites for oviposition^17^, why *L. delicatula* oviposits on non-living materials remains to be explained. The basis for oviposition preferences is not always easy to empirically verify^31^. Some *L. delicatula* researchers think that females lay most of their eggs in the vicinity of where they fed to fatten up and become reproductively mature because it’s harder for them to fly once they’ve gained so much weight^33^. Alternatively, as is evident in some insects and mite species^31,34^, *L. delicatula* may be following a strategy where females do not necessarily oviposit on suitable hosts to make it harder for predators to find their eggs and early instars.

The ability of *L. delicatula* to survive, develop and produce egg masses with or without *A. altissima* may be due to the presence of multiple, suitable host plant species in the enclosures. It is not uncommon for generalist feeders to require diet mixing to acquire the necessary nutrients for development and reproduction^35,36^, and *L. delicatula* appears to demonstrate better fitness when provided multiple suitable host plants. While they can be reared from nymphs to adults on a single species such as *V. vinifera* or *A. altissima*, without diet mixing, adults may not produce eggs. For example, *L. delicatula* that were reared exclusively on *A. altissima* or *V. vinifera* failed to produce egg masses, whereas their counterparts that received a combination of both did (Tracy Leskey, USDA/ARS, personal communication).

Although *L. delicatula* developed more slowly without access to *A. altissima*, the offspring from eggs produced by these adults did not suffer ongoing fitness effects, which could have implications for the spread of this pest in the United States. While range expansion of *L. delicatula* is likely facilitated by the prevalence of *A. altissima*, other hardwood tree species can serve as suitable hosts to support development and reproduction. It’s possible that in a warmer southern climate, adults that develop more slowly in a region without *A. altissima* may have time to catch up and lay as many egg masses as they would have if there was access to *A. altissima*. Overall, our findings suggest that managers need to be aware that even in the absence of *A. altissima* in the landscape, several hardwood host trees can be utilized by *L. delicatula* to develop and reproduce.

## Data Availability

All relevant data are within the paper.
